# A RANDOMISED FEASIBILITY STUDY TO REDUCE SITTING IN OLDER ADULTS UNDERGOING ORTHOPAEDIC SURGERY

**DOI:** 10.1093/geroni/igz038.3127

**Published:** 2019-11-08

**Authors:** Justin A Aunger, Colin T Greaves, Evans A Asamane, Edward T Davis, Anna C Whittaker, Carolyn A Greig

**Affiliations:** 1 University of Birmingham, Birmingham, West Midlands, United Kingdom; 2 The Royal Orthopaedic Hospital NHS Foundation Trust, Birmingham, West Midlands, United Kingdom; 3 University of Stirling, Stirling, Scotland, United Kingdom; 4 School of Sport, Exercise and Rehabilitation Sciences, University of Birmingham, Birmingham, West Midlands, United Kingdom

## Abstract

Osteoarthritis is a prevalent condition in older adults that causes many patients to require a hip or knee replacement in order to improve quality of life and reduce pain. Reducing sedentariness prior to surgery may aid in improving physical function and post-operative outcomes. Thus, we performed a pragmatic randomised controlled feasibility study with 2:1 allocation into intervention or usual care in Birmingham, UK. The intervention involved multiple techniques to reduce sedentary behaviour, including motivational interviewing and action planning. The primary outcome was feasibility, assessed using mixed methods. We included exploratory measures to inform a future definitive trial, such as ActivPal3 objective measurement of physical activity and sedentariness, Short Physical Performance Battery (SPPB), Basic Psychological Needs questionnaire, and serum cardiometabolic biomarkers. Assessments were at baseline, one-week pre-surgery, and six-weeks post-surgery. We recruited 35 participants aged 64-87 years eight weeks before hip or knee arthroplasty. The study was found to be feasible with some modifications. Within-group comparisons showed that the intervention group significantly improved their physical function (SPPB) score by 0.71 points (95% CI 0.07 to 1.36, p=0.032), a clinically significant increase. Additionally, those in the intervention group retained to post-surgery trended towards a mean sedentary time reduction of 66 min.d-1 (p=0.497). In this older mobility-limited surgical population it is feasible to use behavioural techniques to displace sedentary time to activity. Future trials should further assess the effect of reducing sedentariness on health in larger samples of older adults with osteoarthritis undergoing orthopaedic surgery.

